# Using Small-Angle Scattering Data and Parametric Machine Learning to Optimize Force Field Parameters for Intrinsically Disordered Proteins

**DOI:** 10.3389/fmolb.2019.00064

**Published:** 2019-08-13

**Authors:** Omar Demerdash, Utsab R. Shrestha, Loukas Petridis, Jeremy C. Smith, Julie C. Mitchell, Arvind Ramanathan

**Affiliations:** ^1^Biosciences Division, Oak Ridge National Laboratory, Oak Ridge, TN, United States; ^2^University of Tennessee/Oak Ridge National Laboratory Center for Molecular Biophysics, Oak Ridge, TN, United States; ^3^Department of Biochemistry and Cellular and Molecular Biology, University of Tennessee, Knoxville, TN, United States; ^4^Computational Sciences and Engineering Division, Oak Ridge National Laboratory, Oak Ridge, TN, United States; ^5^Data Science and Learning Division, Argonne National Laboratory, Lemont, IL, United States

**Keywords:** intrinsically disordered proteins, machine learning, optimization, force-field parameters, molecular dynamics

## Abstract

Intrinsically disordered proteins (IDPs) and proteins with intrinsically disordered regions (IDRs) play important roles in many aspects of normal cell physiology, such as signal transduction and transcription, as well as pathological states, including Alzheimer's, Parkinson's, and Huntington's disease. Unlike their globular counterparts that are defined by a few structures and free energy minima, IDP/IDR comprise a large ensemble of rapidly interconverting structures and a corresponding free energy landscape characterized by multiple minima. This aspect has precluded the use of structural biological techniques, such as X-ray crystallography and nuclear magnetic resonance (NMR) for resolving their structures. Instead, low-resolution techniques, such as small-angle X-ray or neutron scattering (SAXS/SANS), have become a mainstay in characterizing coarse features of the ensemble of structures. These are typically complemented with NMR data if possible or computational techniques, such as atomistic molecular dynamics, to further resolve the underlying ensemble of structures. However, over the past 10–15 years, it has become evident that the classical, pairwise-additive force fields that have enjoyed a high degree of success for globular proteins have been somewhat limited in modeling IDP/IDR structures that agree with experiment. There has thus been a significant effort to rehabilitate these models to obtain better agreement with experiment, typically done by optimizing parameters in a piecewise fashion. In this work, we take a different approach by optimizing a set of force field parameters simultaneously, using machine learning to adapt force field parameters to experimental SAXS scattering profiles. We demonstrate our approach in modeling three biologically IDP ensembles based on experimental SAXS profiles and show that our optimization approach significantly improve force field parameters that generate ensembles in better agreement with experiment.

## 1. Introduction

Our understanding of classical structure-function paradigm of proteins was first established by recognizing a unique three-dimensional (3D) structure of specific amino acid sequence (Anfinsen, 1973). However, in the late '90s, it was reported that many proteins remain natively unfolded while biologically active (Wright and Dyson, 1999). Such intrinsically disordered proteins or regions (IDPs/IDRs) do not fold autonomously into stable 3D structures; however, they may possess short-transient secondary structure (Uversky, 2011; Das and Pappu, 2013; Latysheva et al., 2015). IDPs typically have an abundance of charged and polar residues while lacking hydrophobic groups. In addition, a recent study suggests IDPs, even with a low net charge, and high hydrophobicity, possess extended conformations in water (Riback et al., 2017). The 3D structure of IDPs is specifically influenced by their sequence, e.g., a linear sequence patterning of oppositely charged residues was found to govern the conformational dimension in polyampholytic IDPs (Das and Pappu, 2013).

Despite the interconverting ensemble of conformations and absence of structured region, IDPs play a vital role in many cell physiology, such as signal transduction and transcription (Habchi et al., 2014; Latysheva et al., 2015; Wright and Dyson, 2015; Mollica et al., 2016). Interest in IDPs also stems from their association with multiple diseases, such as cancers [p53 (Wells et al., 2008) and HPV (Uversky et al., 2006)], diabetes, cardiovascular, and neurodegenerative disorders (e.g., Alzheimer's and Parkinson's diseases) (Uversky et al., 2008; Knowles et al., 2014). Therefore, IDPs not only exemplify a new paradigm for understanding disorder-function relationships but also provide insights on pathological mutations that can lead to serious human diseases (Latysheva et al., 2015).

Nuclear magnetic resonance (NMR) spectroscopy (Wells et al., 2008; Pérez et al., 2009, 2013; Robustelli et al., 2012; Jensen et al., 2014; Arai et al., 2015; Lee et al., 2016; Arbesü et al., 2017), single-molecule Förster resonance energy transfer (smFRET) (Hofmann et al., 2012; Fuertes et al., 2017), cryo-electron microscopy (cEM) (Busch et al., 2015; Levine et al., 2015) and small-angle X-ray scattering (SAXS) (Wells et al., 2008; Receveur-Bréchot and Durand, 2012; Arbesü et al., 2017; Fuertes et al., 2017; Riback et al., 2017; Drulyte et al., 2018) are widely being used to study the disordered structures of IDPs. However, they lack a complete atomic or molecular description of disorder due to instrumental resolution and the ensemble-averaged nature of the measurements, which present a steep challenge to the unambiguous interpretation of the measurements (Fuertes et al., 2017; Kosciolek et al., 2017; Best et al., 2018; Drulyte et al., 2018; Riback et al., 2018). Therefore, molecular dynamics (MD) simulations are often combined with experiments for determining the ensemble of 3D structures of IDPs (Huang et al., 2017).

At the heart of running atomistic molecular dynamics (MD) simulations is a set of empirical potential energy functions from which forces are derived for characterizing the time evolution of a system (typically a protein, or a set of proteins, or other bio-molecules) (Karplus and McCammon, 2002). These potential energy functions are typically referred to as a force field (FF). The last four decades of FF development have been critical in enabling studies of bio-molecular systems in the context of ligand binding, enzyme reactions, protein folding/misfolding and other complex biological phenomena, such as self-assembly (Karplus, 2002).

Current FFs for proteins and other bio-molecules are mature in the sense that they have been rigorously validated for benchmark systems, have an underlying methodology for parameterization, and are being continuously improved upon as discrepancies between simulation results and experimental physical observables arise (Lopes et al., 2015). These deficiencies become particularly noticeable with current advances in sampling ability of MD on modern computer hardware and algorithmic improvements in the software, enabling limitations in sampling to be ruled out as the deficiency (Tiwary et al., 2015). One notable deficiency of standard, pairwise additive force fields is in their ability to correctly capture the experimentally observed properties of intrinsically disordered proteins (IDP) and partial disorder. While empirical force fields have demonstrated a high degree of success in reproducing experimentally derived physical properties of globular proteins, which are characterized by a few relevant, compact conformations, they are deficient in capturing the many transient conformational states and corresponding free energy minima characteristic of IDPs (Huang and MacKerell, 2018). This is best demonstrated in the tendency of empirical force fields to predict a small set of overly compact conformations, in contrast to experimental prediction of a large ensemble of more extended, less compact conformations where the protein interacts much more with solvent (Nettels et al., 2009; Best et al., 2014; Piana et al., 2014, 2015; Skinner et al., 2014). Indeed, this observation, as well as hydration free energy calculations on small molecules being observed to be too unfavorable (Shirts et al., 2003; Shirts and Pande, 2005) compared with experiment, have pointed to standard force fields being excessively solvophobic.

These observations have led researchers to tune the non-covalent energetic parameters in an effort to create a more balanced picture of protein-water interactions. While it could be argued that more complicated functional forms may be necessary, it is highly desirable to be able to preserve the current simple functional forms if possible, given their history of success in capturing an array of biophysical phenomena of interest, and their easy implementation on GPU and other high-performance platforms.

Efforts at rehabilitating FFs for use with IDP/IDR have focused on adjustment of short-ranged non-covalent contributions to protein-water interactions through tuning of van der Waals energetics, modeled in all cases by a Lennard-Jones potential with a 6–12 functional form (Best et al., 2014; Piana et al., 2015; Robustelli et al., 2018). In addition to reparameterization of protein-water interactions, closer attention has been paid to the underlying water model, recognizing the advantages of recently parameterized four-site water models, such as TIP4P-Ew (Horn et al., 2004) and TIP4P/2005 Vega and Abascal (2005), over simpler three-site models, such as TIP3P (Best and Mittal, 2010). Given the overly compact nature of simulated IDP, it was also considered natural to reparameterize the side-chain and backbone torsional parameters, and a number of groups have pursued this line of research (Nerenberg and Head-Gordon, 2011; Rauscher et al., 2015; Huang et al., 2017; Song et al., 2017; Robustelli et al., 2018). Reparameterization of torsional potentials is likely necessary for a different reason, namely, the fact that torsional potentials implicitly have a degree of short-ranged non-bonded character. Despite the continuous progress in improving FF accuracy, our ability to recapitulate gross experimental observables, such as neutron reflectivity/ scattering profiles from MD simulations has therefore remained extremely challenging.

For IDPs, small-angle X-ray and neutron scattering (SAXS and SANS, respectively) are ideal experimental methods for investigating the ensemble of IDP structures, as traditional imaging methods, such as X-ray crystallography or nuclear magnetic resonance (NMR), by themselves are not able to resolve the large number of rapidly interconverting structures of which the IDP ensemble is composed Bernado and Svergun (2012); Kikhney and Svergun (2015). Indeed, low-resolution methods, such as SANS/SAXS are ideal for conformationally polydisperse systems, such as IDP whose conformational ensemble is very large and consists of structures that are rapidly interconverting among themselves. SAXS and SANS are able to provide coarse structural information about the structural ensemble, such as compactness and overall size and shape. Due to the fact that the SAXS/SANS scattering intensities constitute an average over many different structures, these methods must be complemented by additional higher-resolution experimental data, such as NMR observables (Grishaev et al., 2005; Marsh et al., 2007; Marsh and Forman-Kay, 2009; Wang et al., 2009; Schwieters et al., 2010), or simulation-based methods (Bernado et al., 2007; Pelikan et al., 2009; Yang et al., 2010; Rozycki et al., 2011) to elucidate the structures of which the ensemble is composed. Therefore, given the important role of MD simulations as a complement to the interpretation of SAXS/SANS data, it is imperative that the underlying force field be accurate.

**Figure 1:**
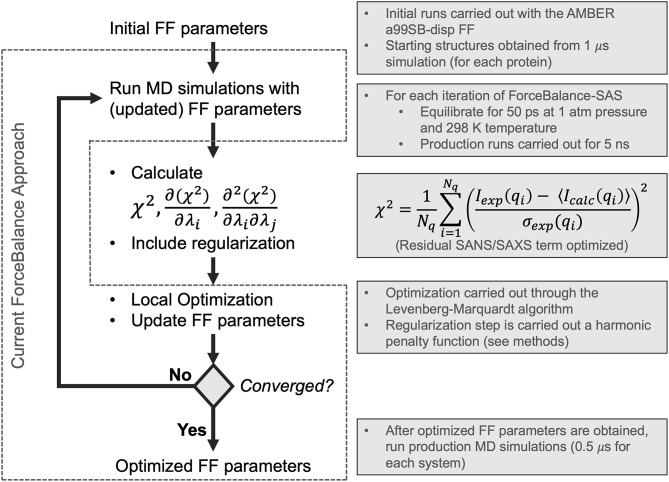
Flowchart depicting the ForceBalance-SAS algorithm. An initial set of parameters is input, followed by MD simulation and calculation of ensemble-averaged small-angle scattering intensities. After the simulation stops, the residual between the simulated and experimental scattering intensities is calculated, along with the gradients and Hessians of the residual. If the desired convergence criteria are met, the algorithm stops, and the new force field parameters are output; if not, optimization is performed, a new set of parameters are obtained, and a simulation with the updated parameters are performed, completing the cycle. The current implementation of the ForceBalance (Wang et al., 2014) approach is demarcated from our approach using dotted lines.

Here, we studied three IDPs with varying molecular weight and different charge-hydrophobicity characteristics (see Figure 1A): RS-peptide (24 residues), PaaA2 (63 residues), and SH4UD (95 residues). RS-peptide is highly charged IDR without any structured region in Serine/arginine-rich proteins, such as serine/arginine-rich splicing factor 1 (SRSF1) and plays a significant role in RNA metabolism, including transcription, RNA splicing and RNA export (Xiang et al., 2013). The phosphorylation of serine residues in RS repeats regulates peptide's interaction and subcellular localization, whereas it undergoes several cycles of phosphorylation and dephosphorylation during splicing (Xiang et al., 2013). PaaA2 is the antitoxin domain of toxin-antitoxin (TA) module in the human pathogen *E. coli* O157, which neutralizes the toxin domain such that TA module copes with different sources of stress (Sterckx et al., 2014, 2016). The TA module is also associated with the establishment of persister phenotype and virulence mechanisms (Sterckx et al., 2016). It has two preformed helices connected by a flexible linker in the absence of a binding partner, however is, classified as IDP due to a high degree of conformational flexibility from SAXS and NMR studies (Sterckx et al., 2014). Proto-oncogene non-receptor human tyrosine kinase c-Src is a multi-domain protein (Tatosyan and Mizenina, 2000; Pérez et al., 2009) that encompasses an N-terminal IDR containing the Src homology 4 (SH4) and unique (U) domains hereafter refer as SH4UD. Several studies suggest the high activity of the c-Src kinase in a wide variety of human cancers, such as colon, breast, pancreas, and brain (Wheeler et al., 2009). The phosphorylation in SH4UD induces a global electrostatic perturbation forcing c-Src kinase to untie from the membrane (Pérez et al., 2009).

In this work, we have implemented a method to optimize FF parameters against experimental SAXS and SANS intensities in ForceBalance (Wang et al., 2014)—these observables can be understood as ensemble-averaged properties with derivable gradients and Hessians with respect to force field parameters. Starting with the most recent and comprehensive reparameterization of an IDP force field (Robustelli et al., 2018) from the D. E. Shaw research group, we optimized the water and protein backbone Lennard-Jones σ and ϵ, as well as the barrier heights of protein backbone torsions, as was done in their study. We sought to determine whether we could systematically improve on the parameters they had derived, as our initial set of parameters was their optimized IDP force field named a99SB-disp. We found that through our systematic reparameterization using ForceBalance that we could achieve improved agreement with experimental SAXS profiles for 3 systems: RS-peptide, PaaA2, and SH4UD. We will henceforth refer to our version of the algorithm as *ForceBalance-SAS* (small-angle scattering). A key advantage of our approach is that nearly any experimental observable can be encoded as an ensemble-averaged property, for which analytic gradients and approximate Hessians with respect to force field parameters that are being optimized can be obtained.

## 2. Methods

### 2.1. Parameter Optimization With ForceBalance-SAS

ForceBalance-SAS parameterization proceeds through an iterative non-linear least-squares minimization of the squared residual between experimental and calculated properties using analytical gradients and approximate Hessians (Gauss-Newton approximation whose term consists of a product of first derivatives) with respect to a set of FF parameters. A flowchart illustrating our approach is shown in Figure 1. Each iteration consists of a MD simulation with the current set of FF parameters, followed by a calculation of the objective function, gradient, and approximate Hessian (at the current set of FF parameter values), and an optimization step using Levenberg-Marquardt algorithm (Levenberg, 1944; Marquardt, 1963) followed by a regularization to avoid overfitting.

The Levenberg-Marquardt algorithm is used, because it is both gradient- and Hessian-based. Moreover, if the initial parameters are far from the local minimum, it is able to converge faster than the Gauss-Newton algorithm. Lastly, the Levenberg-Marquardt algorithm is ideal due to its intrinsic ability to incorporate an adaptive trust radius (Dennis et al., 1981; More and Sorensen, 1983), effectively enabling the algorithm to change the size of the step according to how well the objective function was improved in the previous step, as shown in the following equation framed in the context of the fitting task presented in this work:

(1) $$\left(J^{T}J+\gamma I\right)\delta =J^{T}\left(A^{exp}-\langle A^{calc}\left(\lambda \right)\rangle \right),$$

and

(2) $$\begin{array}{l} J_{ij}=\frac{\partial A_{i}^{calc}}{\partial \lambda_{j}}. \end{array}$$

In the above equation, **A^exp^** is the set of experimentally measured observables, 〈**A^calc^**〉 is corresponding calculated set of ensemble-averaged observables, λ are the parameters (here, FF parameters) whose values we are optimizing, δ is the step taken at the current step of the optimization, and γ is the parameter controlling the adaptive trust radius. In this work, the initial trust radius was set to 1.0, which is larger than the default of 0.1 in the standard ForceBalance approach. A minimum trust radius of 0.05 was allowed (the default in standard ForceBalance is 0.0). An adaptive damping factor controlling how much the trust region can vary from the initial value was set to the default value used in ForceBalance of 0.5. Regularization is achieved by means of a harmonic penalty function that constrains FF parameters to a physically reasonable range of values as follows:

(3) $$\begin{array}{l} R\left(\lambda \right)=\frac{\lambda^{2}}{\alpha^{2}}, \end{array}$$

where *R*(λ) is the harmonic penalty function, λ is the FF parameter, and α corresponds to the radius within which the parameter value can vary. In this work, α is determined by ForceBalance automatically according to the magnitudes of λ, and were 0.0529177, 2.4784, and 96.4853 for van der Waals σ, van der Waals ϵ, and torsional barrier heights, respectively. If convergence criteria are met, the algorithm stops and the optimized FF parameters are output. If not, the cycle continues with a simulation at the new set of parameters.

Our method rests on the ability of ForceBalance-SAS to directly optimize a set of FF parameters with respect to the experimental SAXS and SANS scattering intensities. Any condensed phase observable can be calculated from rigorous statistical mechanical principles. In the isobaric-isothermal ensemble, the ensemble-averaged observable 〈*A*〉 (in our specific case, 〈*I*(*q*)〉, the small-angle scattering intensity—described in Equation 6), for all experimentally observed scattering vectors, *I*(*q*) for a given set of FF parameters λ is:

(4) $$\begin{array}{l} {\langle A\rangle }_{\lambda }=\frac{1}{Q\left(\lambda \right)}\int A\left(r,V,\lambda \right)\exp\left(-\beta \left(E\left(r,V,\lambda \right)+PV\right)\right)dRdV, \end{array}$$

where *Q*(λ) = ∫exp(−β(*E*(*r, V*, λ) + *PV*) is the isothermal-isobaric partition function. Here, *E* is the potential energy, β is $\frac{1}{k_{B}T}$, *T* represents the temperature, *P* is the pressure, and *V* is the volume. In practice, 〈*A*〉 is not evaluated through a direct integration of Equation (4), but rather is sampled numerically by MD assuming ergodicity. Analytic gradients of properties *A* with respect to FF parameters λ can be obtained by analytically differentiating Equation (4):

(5) $$\begin{array}{l} \frac{\partial {\langle A\rangle }_{\lambda }}{\partial \lambda }={\langle \frac{\partial A}{\partial \lambda }\rangle }_{\lambda }-\beta \left({\langle A\frac{\partial E}{\partial \lambda }\rangle }_{\lambda }-{\langle A\rangle }_{\lambda }{\langle \frac{\partial E}{\partial \lambda }\rangle }_{\lambda }\right). \end{array}$$

The above terms are calculated for each value of *I*(*q*) in the experimental (and simulated) scattering profile. Thus, the primary objective of ForceBalance-SAS is to improve the agreement between experimental and calculated SAXS intensities by minimizing the following residual term:

(6) $$\begin{array}{l} \chi^{2}=\frac{1}{N_{q}}\overset{N_{q}}{\underset{i=1}{\sum }}(\frac{I_{exp}\left(q_{i}\right)-\langle I_{calc}\left(q_{i}\right)\rangle }{\sigma_{exp}\left(q_{i}\right)}{)}^{2}, \end{array}$$

where *I*_*exp*_(*q*_*i*_) and *I*_*calc*_(*q*_*i*_) are the experimental and calculated intensities, respectively, at a given wavenumber *q*_*i*_, σ_*exp*_(*q*_*i*_) is the experimental error in the measurement of *I*_*exp*_(*q*_*i*_), and *N*_*q*_ is the number of observations of *q*_*i*_ obtained.

While the expression for the gradient of a property with respect to the FF parameters is analytic, gradients of the potential energy with respect to FF parameters are themselves calculated with three-point finite difference using a step size of 10^−9^. In this work the FF parameters λ were the σ and ϵ of protein backbone Lennard-Jones, and the barrier heights of protein backbone torsions. The final simulation parameters were achieved for RS-peptide and PaaA2 after 18 and 4 cycles of ForceBalance-SAS (Figure S1), respectively, which amounted to the desired reduction in χ^2^ of at least 50%.

### 2.2. SAXS/SANS Calculations

The experimental SAXS data for RS-peptide and PaaA2 were taken from (Rauscher et al., 2015) and (Sterckx et al., 2014), respectively. SH4UD SAXS data was provided by Hugh M. O'Neill, which was measured at X-Ray Laboratory, Spallation Neutron Source, Oak Ridge National Laboratory. SAXS/SANS scattering intensities *I*(*q*) were calculated from MD snapshots using the crysol/cryson algorithms in the ATSAS package (Svergun et al., 1995; Franke et al., 2017). Since crysol/cryson are based on use of implicit solvent, it is essential that its parameter modeling the difference in solvation between the protein surface and bulk be optimized. To achieve this, we averaged the coordinates of all snapshots saved for the simulation of each iteration, and then fit the averaged coordinates to the experimental SAXS/SANS to optimize the solvation parameter; this optimization was done internally within crysol/cryson and details of how this is done can be found in (Svergun et al., 1995). This optimized value was used for the calculated SAXS/SANS of each of the snapshots. Since the calculated and experimental SAXS can have different number of *q* points, a spline-based interpolation of the calculated and experimental SAXS/SANS curves was used to match the number of *q* points between the two. Finally, the calculated SAXS/SANS intensities will necessarily have different amplitudes owing to aspects of the experiment not accounted for in the calculation. To match the amplitudes between calculation and experiment, a linear fit was performed between the SAXS/SANS *I*(*q*) profile averaged over all snapshots and the corresponding experimental *I*(*q*). These fitting parameters were then used for the calculated intensities *I*(*q*) of the individual snapshots.

### 2.3. MD Simulations

The initial MD simulations (step 1 of Figure 1) of three systems (RS-peptide, PaaA2, and SH4UD) were conducted using GROMACS 5.1.2 (Van der Spoel et al., 2005; Hess, 2008; Abraham et al., 2015) using newly developed a99SB-disp FF parameter set (Robustelli et al., 2018). The energy of the system was minimized using 1,000 steepest decent steps, which was followed by 1 ns of equilibration using NVT and NPT ensembles. Finally, 1 μs of production runs were performed using the NPT ensemble. The snapshots saved at the end of the 1 μs simulations were further utilized for ForceBalance-SAS optimization.

For each cycle of ForceBalance-SAS, as part of our optimization procedure (step 2 in Figure 1), each protein was then simulated for 5 ns of production at each iteration in the isothermal-isobaric (NPT) ensemble at 1 atm and 298 K, preceded by 50 ps of equilibration. Achieving statistical convergence of the target scattering property is critical. Our choice of 5 ns of production for each iteration of ForceBalance-SAS was determined heuristically by running a single iteration at a range of production lengths from 0.5 ns to 50 ns. Scattering intensity and Kratky curves were calculated for each simulation length. We used the χ^2^ metric (Equation 6 above) to quantitatively evaluate whether the global features of the scattering profiles at various time-windows from the simulation trajectory (50, 25, 10, 5, 2.5, 1, 0.5 ns) were sufficiently captured (see Figure S2). We found that a choice of 5 ns to have better χ^2^ fit to the experimental data and our choice of 5 ns was an expedient compromise between computational cost and accuracy for each cycle of the optimization. Note that the choice of 5 ns production runs was made based prior to the start of the optimization step. We do note that this length of the simulations may affect the overall quality of fits obtained (see Discussion).

Thermostating (in steps 1 and 2 of Figure 1) was performed using GROMACS (Van der Spoel et al., 2005; Hess, 2008; Abraham et al., 2015) modified Berendsen thermostat (Berendsen et al., 1984) with separate coupling of the protein and solvent to a heat bath at 298 K. Initial velocities assigned according to the Maxwell-Boltzmann distribution at 298 K. Barostating was performed with the Parrinello-Rahman method (Parrinello and Rahman, 1981). A 2-fs timestep was used, and covalent bonds between hydrogen and heavy atoms were constrained using the LINCS algorithm (Hess et al., 1997; Hess, 2008). A 12- distance cutoff was used for van der Waals and the real-space component of electrostatics. Long-range electrostatics were calculated using Particle Mesh Ewald (Darden et al., 1993) with a grid spacing of 1.6. Coordinate snapshots were saved every 100 ps. Simulations were run on a GPU-enabled version of Gromacs (v. 2019) on a single node equipped with two Tesla K80s.

### 2.4. Sequence-Structure Property Predictions

Per-residue disorder prediction was performed with the PONDR (Prediction of Natural Disordered Regions; Obradovic et al., 2003) algorithm using the VLXT model whose predictions are based on the integration of predictions made by three different neural networks. We used the web server CIDER (Holehouse et al., 2017) to ascertain relationships between the charged residue content of a sequence and its structural ensemble propensities.

## 3. Results

SAXS and SANS scattering intensities were implemented as force field parameter fitting targets in ForceBalance-SAS. As the intensities are condensed-phase observables, much of the optimization machinery in ForceBalance-SAS was ideal for this purpose and modification to incorporate SAXS/SANS was straightforward. As our initial set of force field parameters, we used the most state-of-the-art IDP-specific force field, a99SB-*disp*, which has been developed and validated using a comprehensive IDP benchmark consisting of a range of protein systems and experimental observables. To have continuity with their work and previous efforts, we optimized the σ and ϵ of the water and protein backbone atoms' Lennard-Jones, as well as the protein backbone torsion barrier heights. Unlike previous efforts, we are able to optimize all of these simultaneously and, importantly, are able to directly target the agreement of calculated and experimental SAXS scattering profiles. This is an ideal experimental target, as it directly reports on how contracted or expanded protein conformations in the ensemble are, a protein property that force fields have notable difficulty in capturing.

### 3.1. ForceBalance-SAS Enables Better Agreement Between Experimental and Simulated Ensembles

**Figure 2:**
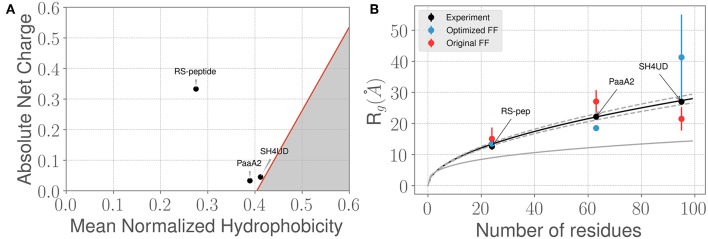
Three prototypical IDP systems chosen for the ForceBalance-SAS approach indicate diverse structural characteristics. **(A)** The mean normalized hydrophobicity vs. the absolute net charge (Uversky) plots indicate that the RS-peptide system is more disordered than the other two systems. The red line is used to mark the boundary between disordered proteins vs. more folded/globular proteins; the gray highlighted area is indicative of the region that is enriched for folded/ globular proteins (Uversky, 2011). **(B)** Comparison of the SAXS determined experimental radius of gyration (R_*g*_) values vs. the R_*g*_ values predicted using simulations from the original FF (red dots) and the optimized FF (blue dots). The theoretical R_*g*_ values predicted from the Flory equation for IDPs (see Results section) is shown in black, along with expected standard deviations (gray dotted lines). The corresponding R_*g*_ values for a globular protein with the same number of amino acid residues is shown for reference (gray solid line). Additional details of the sequence/structural properties of the IDP ensembles considered here are provided in the supporting information.

We chose three prototypical IDP systems that are of biological interest: (1) RS-peptide (Xiang et al., 2013), (2) prokaryotic type II antitoxin module PaaA2 from the human pathogen *E. coli* O157, and (3) the N-terminal regulatory region consisting of the SH4 unique domain (SH4UD) of the C-Src family of non-receptor tyrosine kinases. An examination of the mean hydrophobicity vs. net charge of these three IDP systems, also referred to as the Uversky plots (Uversky, 2011), shows that the RS-peptide system is more disordered than the other two systems (Figure 2A). Not surprisingly, the secondary structural content for the RS-peptide is significantly lower, given that its absolute charge is much higher compared to the other two IDP systems. Indeed from experimental data, such as circular dichroism (CD) and nuclear magnetic resonance (NMR), PaaA2 consists of at least two partially formed α-helices (Sterckx et al., 2014) and SH4UD consists of several transient helices (Pérez et al., 2009; Arbesü et al., 2017). We performed calculations with the CIDER (Classification of Intrinsically Disordered Ensemble Relationships) web server to further parse the sequence-structure relationships based on the fraction of positively and negatively charged residues in the sequence. The diagram of states generated by CIDER shows the propensity of some structure for both PaaA2 and SH4UD (Figure S3), in accord with CD and NMR predictions. RS-peptide presents an interesting case in that it is predicted to be collapsed or expanded, depending on context, but lies very close to the region corresponding to an expanded polyelectrolyte, which is supported by NMR and CD. The experimental observations from NMR and CD are further supported by predictions using the sequence-based prediction method PONDR (Prediction of Natural Disordered Regions), which predicts order for residues 16–35 and 52–75 for PaaA2 and SH4UD, respectively (Figures S4A,B); RS-peptide was too short in length for PONDR to make any prediction.

We next examined how the experimentally determined radius of gyration (R_*g*_) varies with the amino-acid chain length. The experimental R_*g*_ values are obtained through Guinier fits to the scattering profiles. Notably, the experimentally determined R_*g*_ values for the three IDPs aligns closely with the theoretical predictions of ${\text{R}}_{g}^{Flory}$ from the Flory equation: $R_{g}^{Flory}=\left(2.54\pm 0.01\right)\times N^{\left(0.522\pm 0.01\right)},$ where *N* represents the number of amino-acid residues in the IDP of interest. As shown in Figure 2B, the agreement between experimental R_*g*_ and ${\text{R}}_{g}^{Flory}$ is quite remarkable. However, we note that when considering the simulated ensembles, the original a99SB-*disp* FF overestimates the R_*g*_ values for the PaaA2 protein where as the optimized FF underestimates the Rg for the SH4UD ensemble. On the other hand, the ForceBalance-SAS optimized FF overestimates the R_*g*_ values for the SH4UD ensemble, while being close to the experimentally observed R_*g*_ values for the RS-peptide and PaaA2 system. Note that for the SH4UD system, we did not explicitly optimize the FF parameters—we just took the optimized parameters from the PaaA2 simulation and used it to simulate the SH4UD system (see section 3.3).

The Guinier fits to the SAXS profiles for the three IDP systems provide a gross summary of their conformational ensembles; however, the R_*g*_ value by itself does not sufficiently capture all of the information contained in the scattering profiles. We therefore posited that even though the ForceBalance-SAS may underestimate the overall R_*g*_ values, its ability to fit the simulated ensembles to experimentally observed SAXS profiles may be better. To test this hypothesis, we used the χ^2^ metric (Equation 6) to assess the quality of the fit. By optimizing the aforementioned set of force field parameters, we were able to reduce the discrepancy with experiment by a factor of 3.3 and 4.2 for RS-peptide and PaaA2, respectively, where the factor of improvement is simply the ratio of the χ^2^ value obtained with the original parameters to that obtained with the optimized parameters.

**Figure 3:**
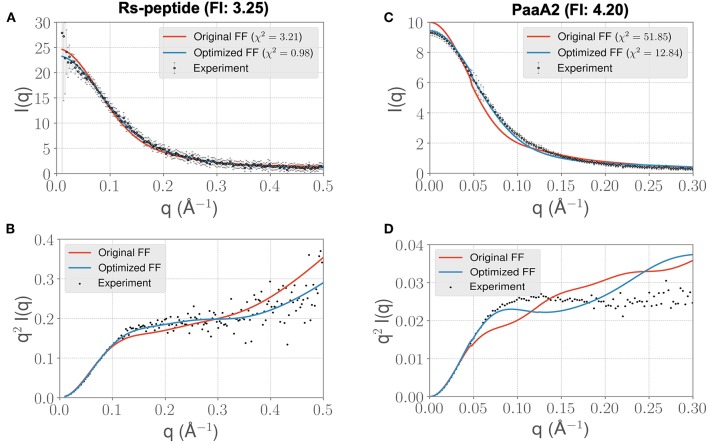
ForceBalance-SAS based simulations generate IDP ensembles that are better fit to the experimental SAXS observables at shorter timescales. **(A)** The scattering profiles for RS-peptide showing the experimental data (black dots with error bars) along with the predicted SAXS scattering profiles from the original FF simulations (red lines) vs. the optimized FF simulations (blue lines). For clarity, the χ^2^ values between the experiment and the respective simulations are shown in the legend. **(B)** The Kratky plot from experiments (black dots), and predicted profiles from simulations (red line corresponding to the original FF, blue line—optimized FF). For clarity, the error bars from the experiments are excluded. **(C,D)** Highlight the same comparison for the PaaA2 system. The factor improvement (FI) in the χ^2^ values between the optimized and original FFs are listed above each protein system.

Visual inspection of the *I*(*q*) vs. *q* profile for RS-peptide (Figure 3A), as well as the Kratky plot (Figure 3B) of *q*^2^*I*(*q*) vs. *q* (Figure 2), reveal more information about the specific aspects of protein structure that have been improved. In general, the lower *q* values report on low-resolution protein behavior, such as how contracted or expanded the structures in the ensemble are, while larger *q* values can report more on finer scale detail. The Kratky plot is useful for quantifying disorder in a polymer chain. For the RS-peptide example, it is clear that the original FF predicts a more disordered ensemble, while both the experiment and the optimized FF based simulations predict some local structure in the ensemble. It is interesting to note that the χ^2^ value has also significantly improved (3.21 with the original FF vs. 0.98 with the optimized FF), indicating that the ensemble from the optimization process has indeed improved the similarity to the experimental data. For the RS-peptide there is evidence of improvement at high *q* values as well, indicating that fine-scale protein-solvent structural details have been improved.

The *I*(*q*) vs. *q* plot for PaaA2 shows marked improvement for the optimized set of parameters in all parts of the profile (Figures 3C,D), and while an improvement is seen for RS-peptide the effect is not as strong (Figure 3A). As can be seen in Figure 3C, improvement is seen at lower *q* values for both RS-peptide and PaaA2, suggesting that the problem with predicting an overly compact ensemble has been remedied.

**Figure 4:**
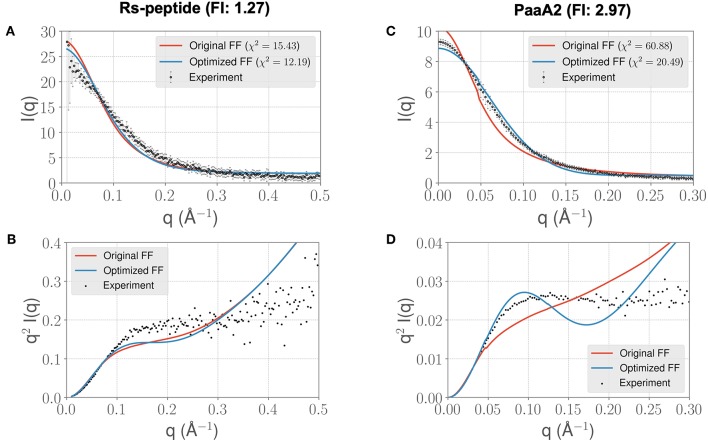
Longer timescale simulations using the ForceBalance-SAS optimized FF parameters preserve improvement with experimentally observed scattering profiles. **(A)** The scattering profiles for RS-peptide showing the experimental data (black dots with error bars) along with the predicted SAXS scattering profiles from the original FF simulations (red lines) vs. the optimized FF simulations (blue lines). For clarity, the χ^2^ values between the experiment and the respective simulations are shown in the legend. **(B)** The Kratky plot from experiments (black dots), and predicted profiles from simulations (red line corresponding to the original FF, blue line—optimized FF). For clarity, the error bars from the experiments are excluded. **(C,D)** Highlight the same comparison for the PaaA2 system. The factor improvement (FI) in the χ^2^ values between the optimized and original FFs are listed above each protein system. Note that the FI in each simulation has decreased compared to the shorter timescales (Figure 3)—however, still preserves the overall trends. It is also notable that the fits of the MD simulations to the longer *q* values are poorer in both cases.

In light of the well-appreciated importance of sampling the rugged conformational landscape of IDPs, we extended our simulations of RS-peptide and PaaA2 using the parameters obtained from the shorter 5-ns simulation lengths to 0.459 and 0.512 μs, respectively. We found that the optimized parameters yield an improvement in χ^2^, albeit more modest than that of the shorter simulation (Figure 4). We note too that the discrepancies between the experimental and simulated ensembles are more apparent at higher *q* ranges, indicating that fine scale interactions are not as well-modeled as global interactions. Nonetheless, this demonstrates that major features of the ensemble that inform the optimization, namely those reflecting large scale interactions, are captured at shorter timescales and are transferrable to longer timescales.

Given the improvements in agreement with experimental observables, it is instructive to ascertain which optimized parameters differed the most from their original values. For both RS-peptide (Tables 1, 2) and PaaA2 (Tables 3, 4), it was the torsional barrier heights that changed the most from their original values. Interestingly, the van der Waals parameters changed little from their original values. This is perhaps expected, given the relatively longer history of attention to balancing solute-solvent, and protein-water, interactions through these terms. This notion is supported by a separate set of calculations where we optimized only the van der Waals parameters for RS-peptide in PaaA2. When only the van der Waals parameters were optimized, the factors of improvement of the χ^2^ values were only 1.98 and 1.3 for RS-peptide and PaaA2, respectively.

**Table 1:** Original and optimized torsion angle parameters for RS-peptide.

**Atom types comprising torsion**	**Original FF**	**Optimized FF**	**% Change**
C–N–CT–C	0.142260	0.145503	2.280
C–N–CT–C	1.40164	1.40177	0.001
C–N–CT–C	2.27610	2.27026	−0.256
C–N–CT–C	0.334720	0.334548	−0.051
H1–CT–C–O	3.34720	3.34905	0.055
H1–CT–C–O	0.334720	0.331802	−0.872
H1–CT–C–OB	3.34720	3.34574	−0.044
H1–CT–C–OB	0.334720	0.334634	−0.026
HB–N–C–OB	8.36800	8.36773	−0.003
HB–N–C–OB	10.4600	10.4603	0.003
N–CT–C–N	0.824250	0.826095	0.224
N–CT–C–N	6.04588	6.05070	0.080
N–CT–C–N	2.00414	2.00474	0.030
N–CT–C–N	0.0799100	0.0797917	−0.148
N–CT–C–N	0.0167400	0.0197590	18.035

*The left-hand label of each row indicates the four atom types of which each torsion is composed. C, backbone carbonyl carbon; N, backbone amide nitrogen; CT, aliphatic carbon (Cα in this context); O, backbone carbonyl oxygen; H1, hydrogen bound to Cα; HB, hydrogen bound to backbone amide nitrogen*.

**Table 2:** Original and optimized Lennard-Jones parameters for RS-peptide.

	**Original FF**		**Optimized FF**			
**Atom type**	**σ**	**ϵ**	**σ**	**ϵ**	**% Change σ**	**% Change ϵ**
C	0.339967	0.359824	0.339966	0.359787	–0.000235359	–0.0104181
H	0.106908	0.0656888	0.106908	0.0656513	–0.000374220	–0.0570937
HB	0.106908	0.0656888	0.106908	0.0657721	–0.000374220	0.126688
N	0.325000	0.711280	0.325000	0.711355	0.000123099	0.0105384
N3	0.325000	0.711280	0.324998	0.711156	–0.000492395	–0.0173983
OB	0.295992	0.878640	0.295992	0.878593	–0.000135163	–0.00539543
O2	0.295992	0.8786401	0.295992	0.878633	0.000135163	–0.000784472
OW-tip4pd	0.316500	0.998989	0.316502	0.998914	0.000505619	–0.00750471

*C, backbone carbonyl carbon; H, hydrogen bound to N-terminal nitrogen; HB, hydrogen bound to backbone amide nitrogen; N, backbone amide nitrogen; N3, N-terminal amine nitrogen; OB, backbone carbonyl oxygen; O2, C-terminal carboxyl oxygen; OW-tip4pd, water oxygen of TIP4P-d model*.

**Table 3:** Original and optimized torsion angle parameters for PaaA2.

**Atom types comprising torsion**	**Original FF**	**Optimized FF**	**% Change**
C–N–CT–C	0.142260	0.144172	1.344
C–N–CT–C	1.401640	1.380281	−1.524
C–N–CT–C	2.276100	2.233383	−1.877
C–N–CT–C	0.334720	0.355767	6.288
H1–CT–C–O	3.347200	3.287138	−1.794
H1–CT–C–O	0.334720	0.356079	6.381
H1–CT–C–OB	3.347200	3.326153	−0.629
H1–CT–C–OB	0.334720	0.355767	6.288
HB–N–C–OB	8.368000	8.378679	0.128
HB–N–C–OB	10.460000	10.438641	−0.204
N–CT–C–N	0.824250	0.845297	2.553
N–CT–C–N	6.045880	6.088597	0.707
N–CT–C–N	2.004140	2.015231	0.553
N–CT–C–N	0.079910	0.068819	−13.880
N–CT–C–N	0.016740	0.023640	41.219

*Refer to the Table 1 legend for an explanation of the atom types*.

**Table 4:** Original and optimized Lennard-Jones parameters for PaaA2.

	**Original FF**		**Optimized FF**			
**Atom type**	**σ**	**ϵ**	**σ**	**ϵ**	**% Change σ**	**% Change ϵ**
C	0.339967	0.359824	0.339979	0.360922	0.0034457	0.30501
H	0.106908	0.0656888	0.106920	0.0654144	0.010798	–0.41769
HB	0.106908	0.0656888	0.106920	0.0656251	0.010957	–0.097042
N	0.325000	0.711280	0.324998	0.710731	–0.00064537	–0.077150
N3	0.325000	0.711280	0.325006	0.711554	0.0018022	0.038575
OB	0.295992	0.878640	0.295998	0.879181	0.0019788	0.061544
O2	0.295992	0.878640	0.295969	0.879181	–0.0079152	0.061544
OW-tip4pd	0.316500	0.998989	0.316512	0.998715	0.0036472	–0.027465

*Refer to the Table 2 legend for an explanation of the atom types*.

### 3.2. ForceBalance-SAS Improves Agreement With NMR Chemical Shift Observables for PaaA2

These observations also led us to the next question: *do the optimized FF parameters allow us to improve agreement with other (independent) experimental observables, such as NMR?* We posited that the improvement in agreement with respect to the gross structural details of the IDPs from SAXS data should also translate to agreement between NMR and MD simulations using the optimized FF. To test this hypothesis, we examined the PaaA2 system in greater detail. While previous work (Sterckx et al., 2014) used both NMR and SAXS data to refine conformational ensembles of PaaA2 using the Flexible-Meccano (Charavay et al., 2012) approach, here we used the optimized FF parameters to recapitulate the NMR chemical shift observables.

**Figure 5:**
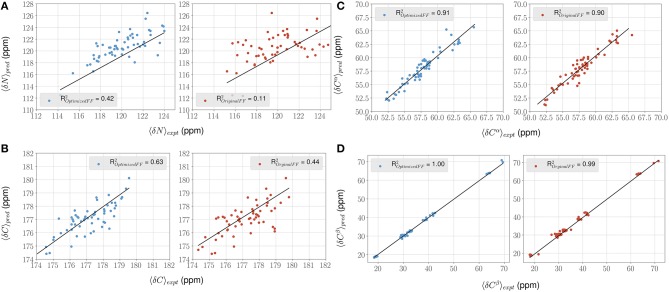
ForceBalance-SAS improves agreement with NMR chemical shift observables for the PaaA2 ensemble. **(A–D)** Panels highlight the comparison between the average experimental (x-axis) chemical shift vs. predicted (y-axis) chemical shift values for N, C^α^, C, and C^β^ atoms, respectively. Predictions from the optimized FF (blue dots) are compared with the original FF (red dots) simulations along with the **R**^**2**^ value for the fits (shown as black lines). Error bars are not highlighted for clarity.

For each conformer in the MD trajectories from the original FF and the optimized FF, we used the program ShiftX2 (Han et al., 2011) to determine the chemical shifts of the backbone atoms: N, C^α^, and C, along with the side-chain: C^β^. We then plotted the agreement between the average experimental chemical shifts with the predicted chemical shifts. As shown in Figures 5A–D, the ForceBalance-SAS optimized FF parameters result in ensembles that are in better agreement with the experimental data, notably for C^α^ and C^β^ atoms. The agreement for the backbone Nitrogen atoms is also significantly improved compared to the original FF, indicating that our approach results in ensembles that better agree with NMR data. Further, for each of the atom types, a non-parametric bootstrap test (*p*-values) for significance also indicated that these correlations are significant (Table 5).

**Table 5:** Summary of the statistical significance in comparing NMR observed chemical shifts with the FF parameters (original and optimized) for PaaA2 system.

	**Original FF**			**Optimized FF**		
**Atom type**	**R^**2**^**	**Standard error**	***p*-value**	**R^**2**^**	**Standard error**	***p*-value**
N	0.11	0.123	1.31E-05	0.42	0.072	1.23E-14
C^α^	0.84	0.056	5.67E-27	0.91	0.039	5.68E-35
C^β^	0.99	0.009	4.52E-72	1.00	0.005	5.53E-85
C	0.44	0.108	9.64E-10	0.63	0.090	5.42E-14

*These were calculated using the sckit.learn package (Pedregosa et al., 2011; Buitinck et al., 2013)*.

**Figure 6:**
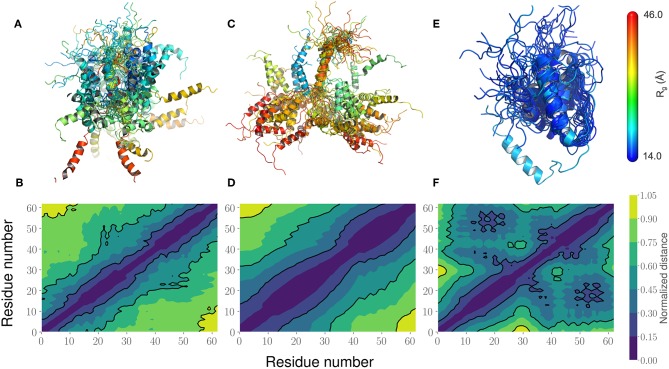
Comparison of the PaaA2 Ensembles determined from experiments and simulations highlight regions of long-range interactions between the two α-helices. **(A)** A cartoon depiction of the PaaA2 ensemble determined using SAXS and NMR techniques using Flexible-Meccano. **(B)** Shows the normalized mean distance matrix showing the various interactions between residues; shades of blue indicate proximity in the chain—implying the increased likelihood of interactions. **(C)** Cartoon representation of the PaaA2 ensemble from the original FF along with the **(D)** normalized mean distance matrix. Note that many of the conformations are in the extended state—indicating less likelihood of interactions between the α-helices. **(E)** Cartoon representation from the PaaA2 ensemble from the optimized FF simulation along with the **(F)** normalized mean distance matrix. The conformations generated by the optimized FF are more compact than the other two datasets mainly because the sampling from the optimization runs are limited.

This led us to further examine the generated ensembles. Each ensemble in Figure 6 is colored using the R_*g*_ value corresponding to that conformation. The experimentally determined ensemble (Flexible-Meccano, Figure 6A) shows the presence of large-scale fluctuations in the orientation between the two α-helices. Each conformer in the ensemble is colored using its R_*g*_ value to highlight the nature of compactness (darker shades of red indicate larger R_*g*_, implying less compact states). To better characterize the nature of these fluctuations, we chose to examine the average (normalized) distance matrix for the experimental ensemble (Figure 6B). This provides us a qualitative measure of the long-range interactions between specific regions of the PaaA2 ensemble. The MD simulations from the original FF capture some of the large-scale fluctuations, however is not fully representative of the experimental data (Figure 6C). Notably, within the experimental ensemble, there are some interactions between the two α-helices, which are not represented in the original FF simulations (Figure 6D). Although visually the average distance matrices look similar, the ensemble generated from the MD simulations using the original FF is dominated by mostly extended states (thus de-emphasizing the interactions between the two α-helices). The simulations from the optimized FF, on the other hand highlight mostly compact conformations (Figure 6E). An examination of the distance matrix (Figure 6F) also shows that there are significantly larger number of interactions between the two α-helices and only localized fluctuations in their relative orientations. We posit that this observation may be a consequence of limited sampling of the conformational landscape (~5 ns every iteration of the optimization).

### 3.3. ForceBalance-SAS Optimized FF Parameters Are Partially Transferable at Shorter Timescales

**Figure 7:**
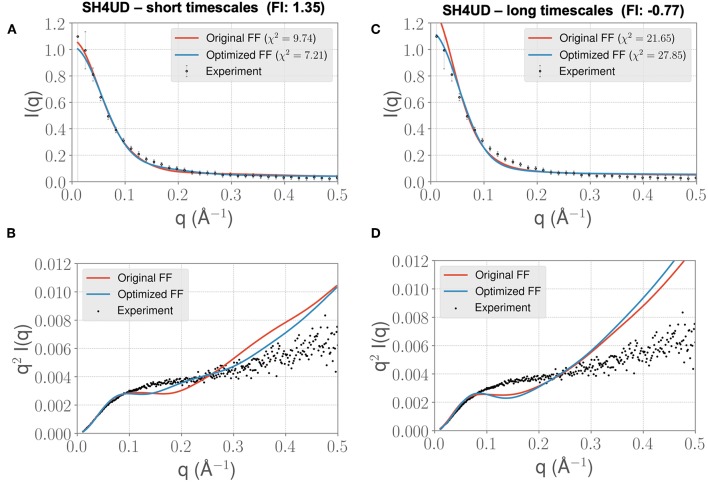
FF parameters learned from the PaaA2 simulations used to simulate the SH4UD IDR improves the fit to experimental SAXS data. Although the factor of improvement (FI) is lower than the other two systems **(A)**, the fit to the experimental data as seen from the Kratky plot **(B)** shows better agreement in the mid-*q* range. This allows us to determine that the parameters learned from one simulation can be used reasonably on other proteins as well. Further fine-tuning may be essential to obtain better fits (especially with solvent-protein interactions). **(C)** and **(D)** highlight the same information as in **(A)** and **(B)** but for longer timescales. Note that the factor of improvement has reversed.

We lastly sought to determine whether our optimized parameters would improve the experimental SAXS agreement for an independent test case. We hypothesized that an appropriate test case would be a protein with a similar charge/hydrophobicity (Uversky) profile, as this has been shown to predict relative disorder/order. For the training system PaaA2, a protein close on the Uversky plot is SH4UD. For this system, we were able to observe a reduction in χ^2^ from 9.7 to 7.2 (Figure 7A), with improvements in agreement seen in the mid-range to high *q* regions of the Kratky plot (Figure 7B). Note that this simulation (with the PaaA2 FF parameters) was carried out only for 5 ns—corresponding to the same timescales of the optimization cycle. Although the improvement in the χ^2^ value is somewhat limited in the high *q* values, we still observe that the ensembles have a better agreement with the SAXS observables.

However, when we extend the simulations to about 0.3 μs, we find that the agreement between experimental SAXS and the MD ensemble deteriorates (see Figures 7C,D). This observation is significant, given the fact that the PaaA2 ensemble consists of two well-defined α-helices (a feature is mostly well-described by existing FFs) and the SH4UD consists of only transient helices, which are not fully captured at the timescales of our current simulations. Further studies would be necessary to validate these simulations (and the transferability of the FF parameters at longer timescales) against available experimental data.

## 4. Discussion

We have presented a proof-of-concept demonstration to optimize a set of FF parameters using small-angle scattering data on a protein-by-protein basis. We started with a few assumptions, including that (1) simulations would be initiated from a single starting structure (for e.g., from an experimental crystal structure), (2) MD simulations would be performed under some equilibrium conditions without necessitating enhanced sampling techniques, such as replica exchange, and (3) longer time-scale simulations (*O*(μ*s*) would not be accessible for all systems of interest. Such assumptions, especially in the context of IDP systems may seem limiting, given that both enhanced sampling and ensemble MD simulation techniques are known to improve the overall ability of MD simulations to “match” experimental observations (Lee and Chen, 2016; Holehouse et al., 2017; Bhattacharya and Lin, 2019). We believe that the optimization scheme outlined here can be extended in a straightforward way for ensemble MD strategies, and it would need some modifications for enhanced sampling strategies. This is a direction that we will pursue in the near future.

The fact that our method seemed to change the torsional parameters much more than the van der Waals is noteworthy. As mentioned previously, the torsional components are covalent energetic degrees of freedom, but also implicitly contain a degree of non-covalent character, given the larger 1-4 separation of the atoms (as opposed to the 1–2 and 1–3 separations for bond stretching and angle bending, which can more definitively be considered purely covalent). It is therefore likely that short-ranged non-covalent energetics that are not explicitly accounted for in typical force field functional forms are being folded into the torsional term.

We note that the fitting procedure used in ForceBalance-SAS improves the agreement with independent observations, such as NMR chemical shifts. NMR chemical shifts represent effective local measurements for conformational changes in an ensemble and provide a powerful technique to characterize IDP/IDR ensembles in the context of their biological function (Pérez et al., 2009; Sterckx et al., 2014; Arbesü et al., 2017). Our optimization procedure takes into account only the SAS measurements. However, by fitting our MD ensembles to SAS curves, we also found that it consequently improved the agreement of local measurements. In the context of modeling IDP/IDR ensembles, our approach therefore represents a complementary approach to using multiple experimental methods to capture atomistic details of these systems. Whereas approaches such as Flexible-Meccano (and other tools) utilize all of the available experimental data to model IDP/IDR ensembles, our iterative approach can be modified to take into account gross structural features first, and then followed by further tuning FF parameters to recapitulate fine-grained features.

We also showed that the optimized FF parameters developed for an IDP could be transferred (in a limited manner) to other IDPs. Although the improvement in agreement between experiments and simulations was only marginal, we were still able to recapitulate some of the finer grained details of the SH4UD ensemble better than the original FF at short simulation length. The parameters that get optimized most likely depend on the amount of sampling carried out at each iteration. While preliminary testing indicated that calculated SAXS profiles appeared to converge at about 5 ns for each iteration, it is likely that this may not hold for all IDP systems of interest, especially those that are larger than the systems studied here. Indeed, the rugged free energy/conformational landscapes of IDP are very different from those of systems such as neat water to which the parent ForceBalance method had been previously applied (Wang et al., 2013, 2014; Laury et al., 2015). Nonetheless, the fact that longer simulations at 100s of nanoseconds performed with parameters obtained from a 5-ns simulation length still show improved agreement of the MD ensemble with the experimental SAXS supports the view that major signatures of the full ensemble are captured and can be optimized against to yield the observed improvement at longer timescales. Further work on the reproducibility of our approach is also needed, especially in the context of benchmark IDP/IDR ensembles that have been recently made available (Varadi et al., 2013). To this end, the effect of the simulation length in ForceBalance-SAS on the resulting parameters will be investigated in the future.

We are endeavoring to enhance this method on a number of fronts. We plan on addressing the sampling issue by deploying this method on more powerful supercomputers so that longer simulations in each cycle of the algorithm are less onerous. We also note that in all cases, the ability to optimize in the higher *q* range was poorer than in the low *q* range, as is best depicted in the Kratky plots. This indicates that in the current regime, we are optimizing global scale interactions better than fine scale interactions. Therefore, it is only natural that a worthwhile objective is to differentially weight the contributions of different q regions to the objective function during the optimization. Furthermore, current work is focused on optimizing FF parameters using the experimental data of multiple protein targets simultaneously, which should enhance the transferability of the optimized parameters. Nonetheless, for those who are interested in detailed simulation studies of specific systems, the current system-by-system approach is useful.

## Data Availability

The datasets generated for this study are available on request to the corresponding author.

## Author Contributions

OD, AR, JM, JS, and LP conceived the project. OD developed methodology, implemented and tested the techniques and ran simulations. US and LP ran simulations. OD and AR contributed analysis tools and analyzed the data. All authors wrote, edited, and approved the manuscript.

### Conflict of Interest Statement

The authors declare that the research was conducted in the absence of any commercial or financial relationships that could be construed as a potential conflict of interest.

## Supplementary Material

Supporting information consists of additional figures for the generated IDP ensembles. The MD simulation datasets, as well as the analysis codes are available upon request.
