# Supplementary material for: Using Small-Angle Scattering Data and Parametric Machine Learning to Optimize Force Field Parameters for Intrinsically Disordered Proteins
Source: Front Mol Biosci. 2019 Aug 13;6:64. doi: 10.3389/fmolb.2019.00064 (PMC6705226; doi:10.3389/fmolb.2019.00064)
Supplement: Supplementary file 1 [file Data_Sheet_1.PDF]

Supporting information for:

# Using small-angle scattering data and parametric machine learning to optimize forcefield parameters for intrinsically disordered proteins


Omar Demerdash[1,2], Utsab R. Shrestha[1,2], Loukas Petridis[1,2], Jeremy C. Smith[2], Julie C. Mitchell[1,2], and Arvind Ramanathan[3,4*]

[1]Biosciences Division, Oak Ridge National Laboratory, Oak Ridge, TN

[2]UT/ORNL Center for Molecular Biophysics, Oak Ridge TN, USA

[3]Computational Sciences and Engineering Division, Oak Ridge National Laboratory, Oak Ridge, TN, USA

[4]Data Science and Learning Division, Argonne National Laboratory, Lemont, IL, USA

*Correspondence: ramanathana@anl.gov


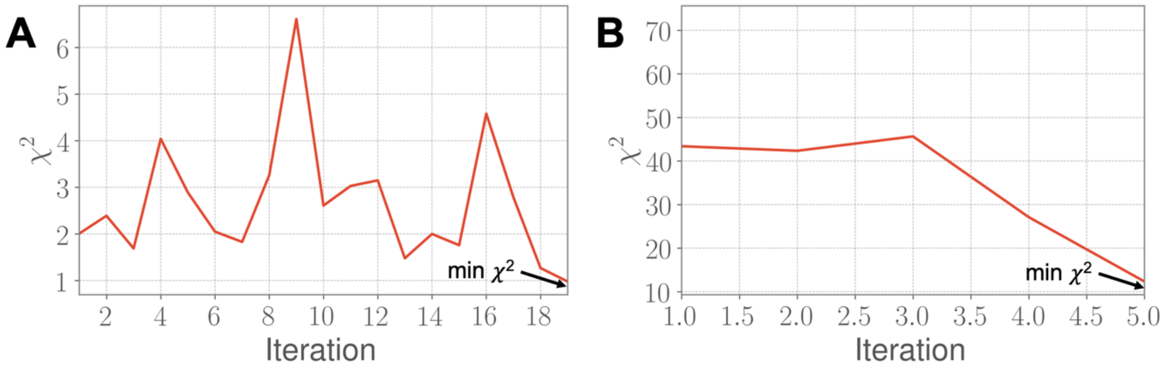


**Figure S1: The optimization procedure of ForceBalance-SAS minimizes the$\chi^2$ metric (eqn. 6 in main text) between experimentally observed and theoretically calculated scattering profiles.** For the RS-peptide system, we see that the FF parameters chosen at the end of the 18th iteration results in the minimum $\chi^2$ value whereas for the PaaA2 system, we need about 4 iterations to find the minimum $\chi^2$ value. Each iteration consists of a run of ForceBalance followed by a production run of 5 ns MD (see main text of details).

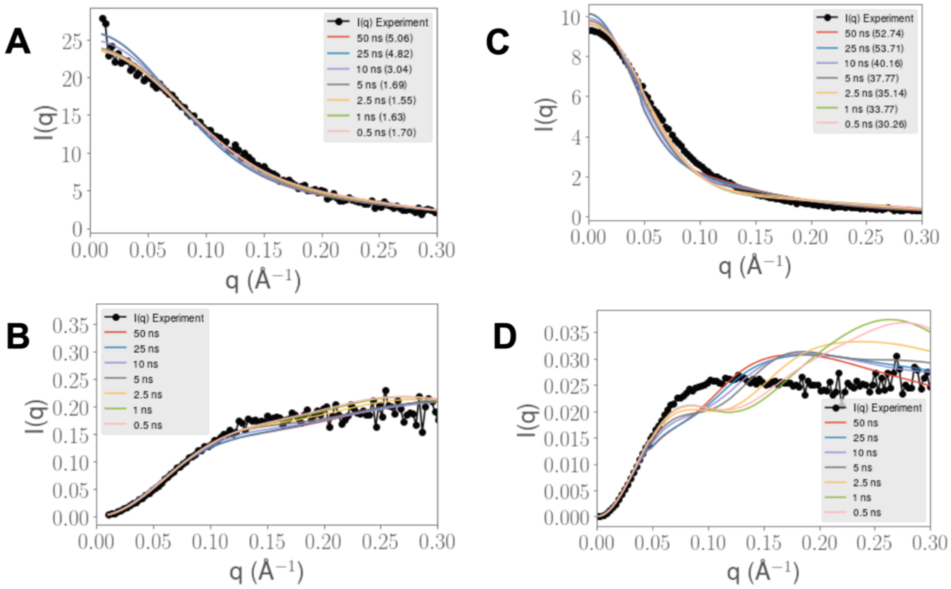


**Figure 2: Simulation lengths and variation of $\chi^2$ metric (eqn. 6 in main text) for (A, B) RS-peptide and (C, D) PaaA2 systems.** Using the original FF parameters, we swept a window size of 50 ns through 0.5 ns and compared the calculated SAS profiles with respect to the experimental data. The $\chi^2$ value within parentheses in panels A and C provide a quantitative view of the dependence between the time window chosen and the fit with the experimental data. Our choice of 5 ns for the production runs in the optimization cycle are based on the tradeoff between computational efficiency and the overall fit obtained with respect to the experimental profiles.

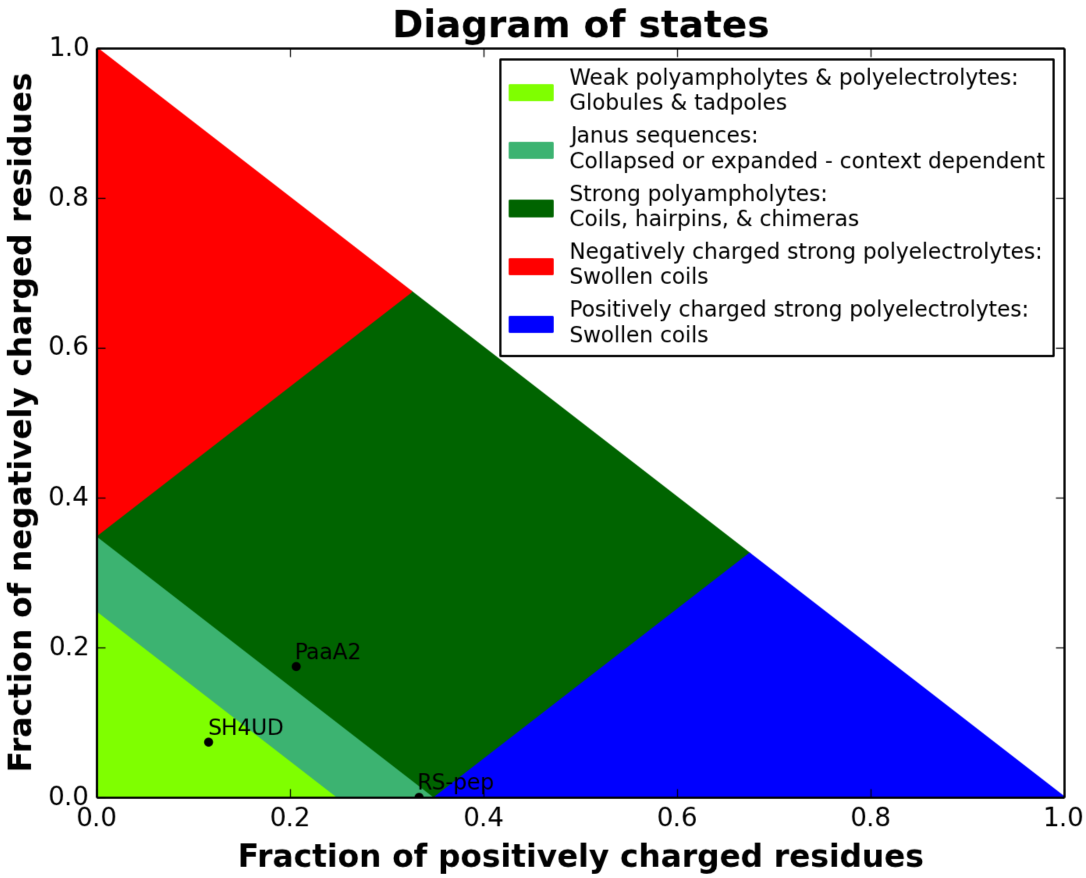


**Figure S3: CIDER (Classification of Intrinsically Disordered Ensemble Relationships) predicted sequence features of RS-peptide, PaaA2 and SH4UD systems chosen for simulations.** The CIDER predictions measure the proportion of positively charged versus negatively charged residues and provides insights into the nature of the overall conformational ensembles that can be characterized. Main text provides additional details about the three systems.

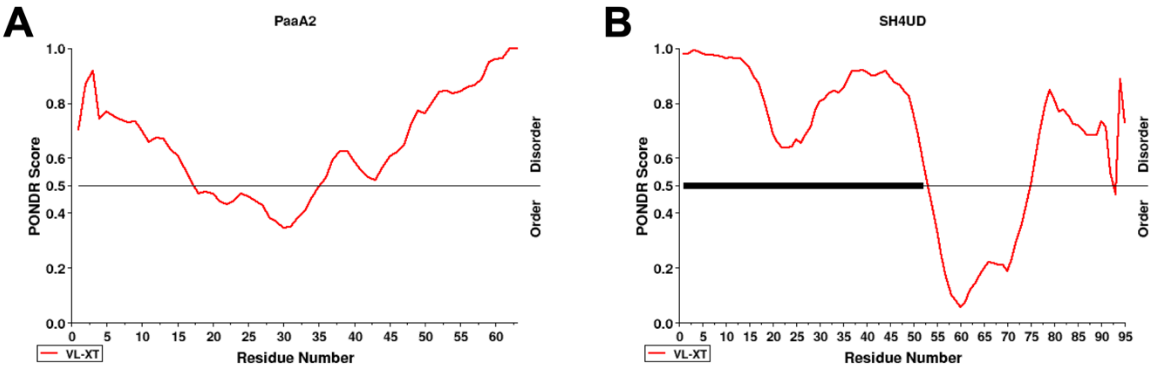


**Figure S4: Disorder prediction at the sequence level for PaaA2 and SH4UD systems using the PONDR (Predictor of Natural Disordered Regions) program using the VLXT predictor.** The sequence for RS-peptide (GAMGPSYGRSRSRSRSRSRSRSRS) is not shown as it is too short for predictions to work effectively. The PONDR score provides a score that identifies where the two systems, namely PaaA2 (KNRALSPMVSEFETIEQENSYNEWLRAKVATSLADPRPAIPHDEVERRMAERFAKMRKERSKQ) and SH4UD (MGSNKSKPKDASQRRRSLEPAENVHGAGGGAFPASQTPSKPASADGHRGPSAAFAPAAAEPKLFGGFNSSDTVTSPQRAGPLAGGSAWSHPQFEK) are disordered. See main text for more details.
